# Spatial Patterns in the Distribution of Hypertension among Men and Women in India and Its Relationship with Health Insurance Coverage

**DOI:** 10.3390/healthcare11111630

**Published:** 2023-06-02

**Authors:** Rajesh Kamath, Helmut Brand, Harshith Ravandhur Arun, Vani Lakshmi, Nishu Sharma, Reshma Maria Cocess D’souza

**Affiliations:** 1 Prasanna School of Public Health, Manipal Academy of Higher Education, Manipal 576104, Karnataka, India; 2 Department of International Health, Care and Public Health Research Institute—CAPHRI, Faculty of Health, Medicine and Life Sciences, Maastricht University, 6211 LK Maastricht, The Netherlands; 3 World Bank, New Delhi 110001, India; 4 Department of Medical Laboratory Technology, Manipal College of Health Professions, Manipal Academy of Higher Education, Manipal 576104, Karnataka, India

**Keywords:** health insurance, hypertension, Ayushman Bharat, health and wellness centers

## Abstract

The present study explores district-level data associated with health insurance coverage (%) and the prevalence of hypertension (mildly, moderately, and severely elevated) observed across men and women as per NFHS 5. Coastal districts in the peninsular region of India and districts in parts of northeastern India have the highest prevalence of elevated blood pressure. Jammu and Kashmir, parts of Gujarat and parts of Rajasthan have a lower prevalence of elevated blood pressure. Intrastate heterogeneity in spatial patterns of elevated blood pressure is mainly seen in central India. The highest burden of elevated blood pressure is in the state of Kerala. Rajasthan is among the states with higher health insurance coverage and a lower prevalence of elevated blood pressure. There is a relatively low positive relationship between health insurance coverage and the prevalence of elevated blood pressure. Health insurance in India generally covers the cost of inpatient care to the exclusion of outpatient care. This might mean that health insurance has limited impact in improving the diagnosis of hypertension. Access to public health centers raises the probability of adults with hypertension receiving treatment with antihypertensives. Access to public health centers has been seen to be especially significant at the poorer end of the economic spectrum. The health and wellness center initiative under Ayushman Bharat will play a crucial role in hypertension control in India.

## 1. Introduction

It is well established that elevated blood pressure (BP) is a public health challenge of significance. Once seen to be a condition of the rich countries, today it is a tall challenge for low- and middle-income countries (LMICs) [1]. India is estimated to have nearly 200 million adults with elevated BP [1]. Elevated BP is a critical determinant of increasing morbidity and mortality from heart disease [2,3,4]. Globally, high systolic BP (SBP) accounts for 10 million deaths and 208 million disability adjusted life years (DALYs) [5]. Approximately one tenth of DALYs can be traced to high SBP, which is responsible for more than half of the DALYs due to ischemic heart disease and stroke [5]. The global prevalence of hypertension (defined as systolic BP ≥ 140 and/or diastolic BP ≥ 90 mm Hg) is increasing, with more than 1 billion people having it [1,6,7]. There has been a near doubling in the gross numbers of adults with elevated blood pressure from 594 million in 1975 to 1.13 billion in 2015, with the bulk of this increase being in LMICs [1]. Evidence from reviews of epidemiological studies of hypertension in India points to a substantial and ever-increasing burden of elevated BP [8,9,10,11]. The increase is across both rural and urban areas. Epidemiological studies of hypertension in India in the 1950s used BP ≥ 160 mm Hg systolic and/or 95 mm Hg diastolic as the standard threshold and estimated an adult prevalence of 1.2 to 4 percent [8]. Subsequent evidence indicated an increase in adult hypertension prevalence in urban areas from 3.0 percent to 4.5 percent in the 1960s to 11.0 to 15.5 percent in the mid-1990s [8]. The same time period saw an increase in adult hypertension prevalence in rural areas from less than 1 percent in the 1960s to 5–7 percent in the 1990s [8,9]. From the 1990s onwards to present date, cross sectional regional studies clearly indicate a rising trend in hypertension prevalence in India across rural and urban geographies. This increase is greater in studies conducted in urban geographies compared to studies conducted in rural geographies [11].

Multicentric studies in India using similar tools indicate that hypertension prevalence is higher in urban geographies than in rural geographies [10]. The adult prevalence of hypertension in India reported in these studies is markedly higher at 25 percent, which is similar to other LMICs and only slightly lower than the prevalence reported in high income countries [6]. However, these studies have limitations: limited geographical generalizability, differing methodologies, differing age groups of study participants, variation in type of BP measuring apparatus used, number of measurements in a day, number of days that BP was measured, method of calculating the final reading, etc. [9,10,11]. Systematic reviews of hypertension epidemiology studies in India at the local and regional levels report a hypertension prevalence of 25 to 30 percent in urban areas and 10 to 20 percent in rural areas [9,10,11] with significant regional variations. A significant probability exists of these rates not being statistically representative for the country. There are few multicentric studies to estimate hypertension prevalence, with lack of geographic coverage and absence of usage of similar tools being the major drawbacks. Even with regard to population coverage, limitations exist, with the study cohorts limited to adult populations in selected rural areas [12], adult populations in both urban and rural areas from selected geographies [13,14,15,16], blue collar workers in industries [17], adults belonging to the socioeconomic middle class [18] and women belonging to the socioeconomically weaker sections of society [19]. These studies show a hypertension prevalence of 20 to 35 percent in adult populations and 15 to 20 percent in rural populations.

Increasing health insurance coverage would be expected to be associated with improvements in health indicators, including hypertension prevalence. It would therefore be natural to expect geographies with higher rates of health insurance coverage to have lower rates of hypertension prevalence and vice versa. Health insurance coverage would be expected to have a negative relationship with hypertension prevalence. In this study, we determine the spatial patterns in the distribution of hypertension among men and women in India and assess its relationship with health insurance coverage.

## 2. Methodology

### 2.1. Study Population and Data Collection

The National Family Health Survey (NFHS) is carried out in all the states and union territories of India. The NFHS gathers population, nutrition, and health data. The fifth survey in the series (NFHS-5) was carried out in 2019–2020. The NFHS is a valuable source of high-quality district level estimates for several critical indicators. It is conducted by the Government of India under the aegis of the Ministry of Health and Family Welfare (MoHFW). The executive agency for the NFHS is the International Institute for Population Sciences (IIPS), Mumbai. The NFHS receives technical assistance from Demographic and Health Surveys Program, ICF, USA, which is supported by USAID, the National AIDS Research Institute (NARI), Pune and the Indian Council of Medical Research (ICMR), New Delhi. The NFHS all India sample size is 610,000 households. This number is expected to provide statistically significant results at the district level: as of August 2022, India has 766 districts for 1.4 billion people. For both rural and urban areas, a two-stage sample design was used. In the first stage in rural areas, villages were the Primary Sampling Units (PSUs). In the second stage, 22 households were chosen by random selection in each PSU. In the first stage in urban areas, Census Enumeration Blocks (CEB) were the Primary Sampling Units (PSUs). In the second stage, 22 households were chosen by random selection in each CEB. In the second stage in both rural and urban areas, a comprehensive mapping and listing of households was completed before households were selected for the survey [20].

### 2.2. Measurement

We carried out secondary data analysis based on the data collected as part of 2019–2021 NFHS-5. The fieldwork for NFHS-5 was executed in two phases. The first phase was from 17 June 2019 to 30 January 2020. The second phase was from 2 January 2020 to 30 April 2021. The data gathering was done by 17 Field Agencies. A total of 636,699 households, 724,115 women, and 101,839 men participated in the exercise [21]. Informed, written consent was received from all participants. Field workers in charge of gathering data were administered adequate training to measure blood pressure accurately. The field manual and the questionnaire for data collection are available online [22]. A portable Omron blood pressure monitor was used to measure blood pressure. Before BP measurement, the participant was required to be seated quietly for more than five minutes. The participant was requested to abstain from consuming tea or coffee and from smoking at the time of their BP measurement. The participant was seated at a table in a relaxed posture with feet on the floor. The preferred site of BP measurement was the left arm. BP was measured thrice with a gap of five minutes between measurements. The accuracy of the data gathered was ensured by field supervisors revisiting a subset of participants selected randomly. All data were transferred every day to the Indian Institute of Population Sciences. A detailed description is given in the field manual [22].

### 2.3. Statistical Analysis

The present study explores district-level data associated with health insurance coverage (%) and the prevalence of hypertension (mildly, moderately, and severely elevated) observed across men and women as per NFHS 5. Spatial analysis was carried out using QGIS 3.26 (Mac version) and GeoDA 1.20.0.8. Firstly, a spatial weight matrix has been calculated to quantify the spatial proximity between each possible pair of regions. Further, the spatial clustering of the characteristics of interest was examined using Local Moran’s I statistic, which measures the spatial autocorrelation and indicates the degree to which data points are similar or dissimilar to their neighbors. The p-value of Local Moran’s I was generated using a randomization test on a Z-score with 999 permutations.

Following this, Univariate Local Indicators of Spatial Association (LISA) measure the correlation of neighborhood values around a specific spatial location and determine the extent of spatial randomness and clustering in the data. This provided LISA cluster maps and significance maps, respectively. The following scenarios are presented on the maps, which are linked to the quadrants of Moran’s I scatter plot:

- Hotspots: regions with high values, with similar neighbors (high–high)
- Cold spots: regions with low values, with similar neighbors (low–low)
- Spatial outliers: regions with high values, with low-value neighbors (high–low)
- Regions with low values but with low-value neighbors (low–high)

Subsequently, spatial correlation analysis was carried out to explore associations between district-level hypertension and health insurance coverage patterns based on findings from NFHS 5.

## 3. Results

### 3.1. Part A: Mildly Elevated Blood Pressure

**Figure 1:**
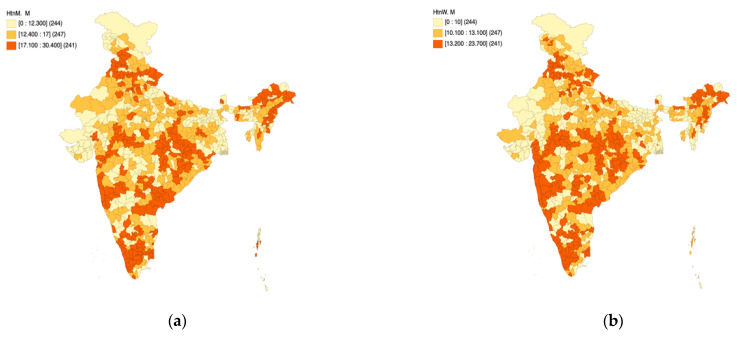
NFHS-5: District level patterns in mildly elevated blood pressure among women (**a**) and men (**b**).

Figure 1a,b presents the spatial quantile maps for mildly elevated blood pressure (%) across women (Figure 1a) and men (Figure 1b) as per NFHS-5 to unravel district-level patterns across the length and breadth of the country. As per the operational definition of NFHS-5, mildly elevated blood pressure is defined as “Systolic: 140–159 mm of Hg and/or Diastolic: 90–99 mm of Hg”. The notations “HtnW. M” and “HtnM. M” have been used on the maps to indicate mildly elevated blood pressure among women and men, respectively.

The color coding is an indicator of spatial patterns, wherein the darker colors indicate a higher percentage of men/women living with mildly elevated blood pressure, and the lighter colors indicate a lower percentage of men/women living with mildly elevated blood pressure. A visual assessment of the maps reveals several dark spots in the country indicating a higher risk of hypertension among its citizens. Over one-third of the districts in the country are likely to face the hypertension-induced multi-faceted burden in the coming years. Based on Figure 1a,b, it is observed that the states of Kerala, Tamil Nadu, coastal Karnataka, and Maharashtra are home to a higher percentage of men/women living with mildly elevated blood pressure. This phenomenon is also observed in parts of northeast India as well. Higher within-state variability is observed in central India. While most parts of Gujarat and Rajasthan appear to be at relatively less risk in the context of hypertension as per NFHS 5, both states need to focus on efforts to continue the ongoing momentum to bring down the burden of mildly elevated blood pressure.

**Figure 2:**
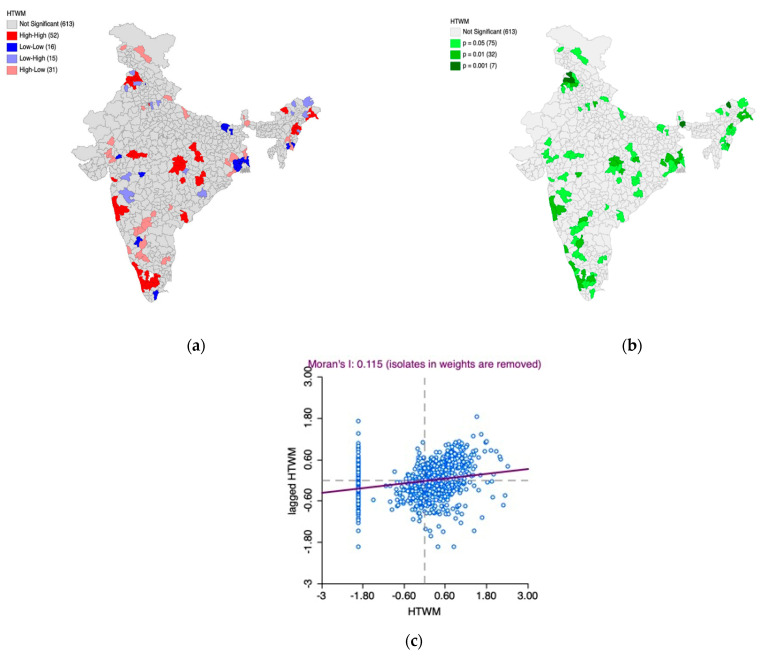
(**a**) Univariate Local Indicators of Spatial Association (LISA) cluster map for the prevalence of mildly elevated blood pressure among women, (**b**) LISA significance map for the prevalence of mildly elevated blood pressure among women, (**c**) Moran’s I scatter plot.

**Figure 3:**
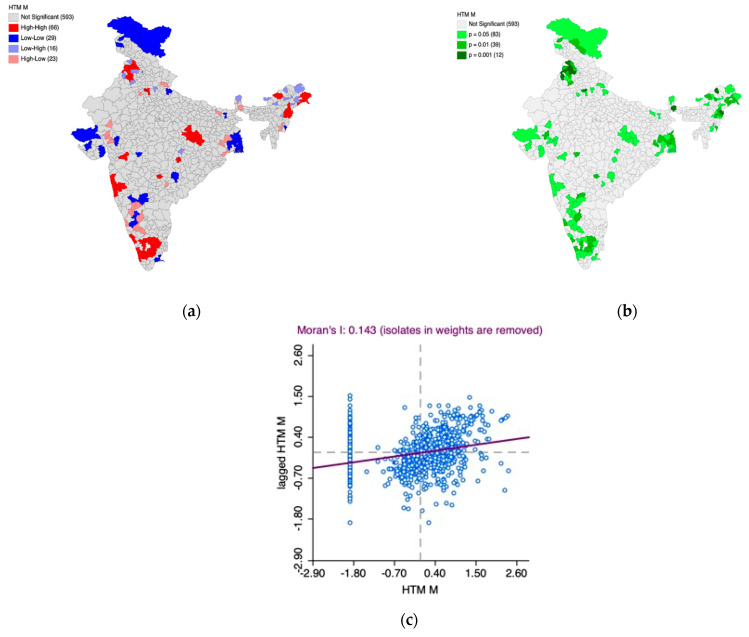
(**a**) Univariate Local Indicators of Spatial Association (LISA) cluster map for the prevalence of mildly elevated blood pressure among men, (**b**) LISA significance map for the prevalence of mildly elevated blood pressure among men, (**c**) Moran’s I scatter plot.

Furthermore, the Univariate Local Indicators of Spatial Association (LISA) cluster map and the LISA significance map, along with Moran’s I scatter plot, was generated to understand the underlying spatial phenomenon associated with the prevalence of mildly elevated blood pressure among women (Figure 2a–c) and men (Figure 3a–c).

Based on Figure 2a–c, we observed 52 hotspot (indicated by the color red) districts scattered across the country with over 16 of them in south India. This means that there are 52 districts in the country with high prevalence of mildly elevated blood pressure among women which are also surrounded by districts with the same phenomenon which is statistically significant as well.

The Moran’s I statistic (I = 0.115, *p* < 0.05) confirms the spatial autocorrelation.

Based on Figure 3a–c, we observed 66 hotspot (indicated by the color red) districts scattered across the country, with over 19 in South India. This means that there are 66 districts in the country with a high prevalence of mildly elevated blood pressure among men, which are also surrounded by districts with the same phenomenon, which is statistically significant.

A majority of these districts are in the western coastal region with a particular focus on the states of Kerala and Tamil Nadu. In addition, most of the cold spots (indicated by the color blue) are located in Jammu and Kashmir, parts of Gujarat and West Bengal. These regions have districts with a lower prevalence of elevated blood pressure among men and are surrounded by the districts with the same phenomenon, which is statistically significant.

The Moran’s I statistic (I = 0.143, *p* < 0.05) confirms the spatial autocorrelation.

### 3.2. Part B: Moderately/Severely Elevated Blood Pressure

**Figure 4:**
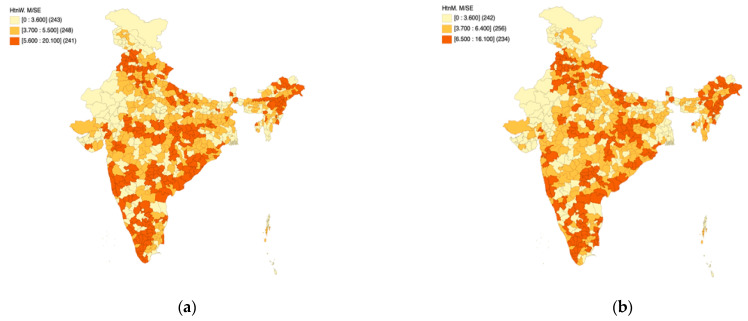
NFHS-5: District level patterns in moderately/severely elevated blood pressure among women (**a**) and men (**b**).

Figure 4a,b presents the spatial quantile maps for moderately/severely elevated blood pressure (%) across women (Figure 4a) and men (Figure 4b) as per NFHS-5 to unravel district-level patterns across the length and breadth of the country. As per the operational definition of NFHS-5, moderately/severely elevated blood pressure is defined as “Systolic: ≥160 mm of Hg and/or Diastolic: ≥100 mm of Hg”. The notations “HtnW. MSE” and “HtnM. MSE” have been used on the maps to indicate moderately/severely elevated blood pressure among women and men, respectively. The color coding is an indicator of spatial patterns, wherein the darker colors indicate a higher percentage of men/women living with moderately/severely elevated blood pressure, and the lighter colors indicate a lower percentage of men/women living with moderately/severely elevated blood pressure. A visual assessment of the maps reveals several dark spots in the country, indicating a higher risk of hypertension among its citizens. Over one-third (241 districts in the case of women and 234 districts in the case of men) of the districts in the country are likely to face the hypertension-induced multi-faceted public health burden given the fact that the maps showcase district-level spatial patterns in moderate/severely elevated blood pressure.

Based on Figure 4a,b, it is observed that the district-level spatial patterns of moderate/severely elevated blood pressure are similar in the context of men and women. While parts of Gujarat, Rajasthan, and Jammu and Kashmir have a relatively low prevalence, the coastal districts in the peninsular region of the country experience higher prevalence (expressed in %). This phenomenon is also observed in northeast India, particularly in parts of Assam. The central part of India appears to have high within-state heterogeneity.

**Figure 5:**
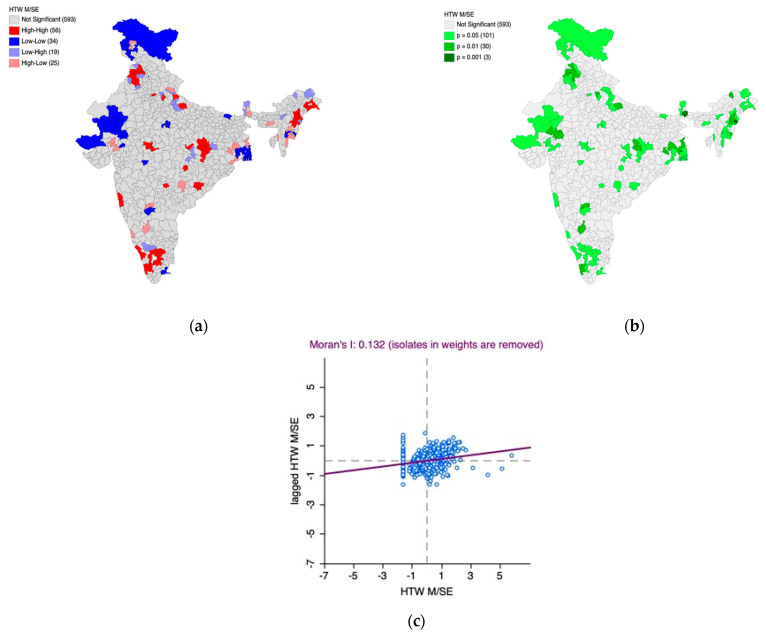
(**a**) Univariate Local Indicators of Spatial Association (LISA) cluster map for the prevalence of moderately/severely elevated blood pressure among women, (**b**) LISA significance map for the prevalence of moderately/severely elevated blood pressure among women, (**c**) Moran’s I scatter plot.

**Figure 6:**
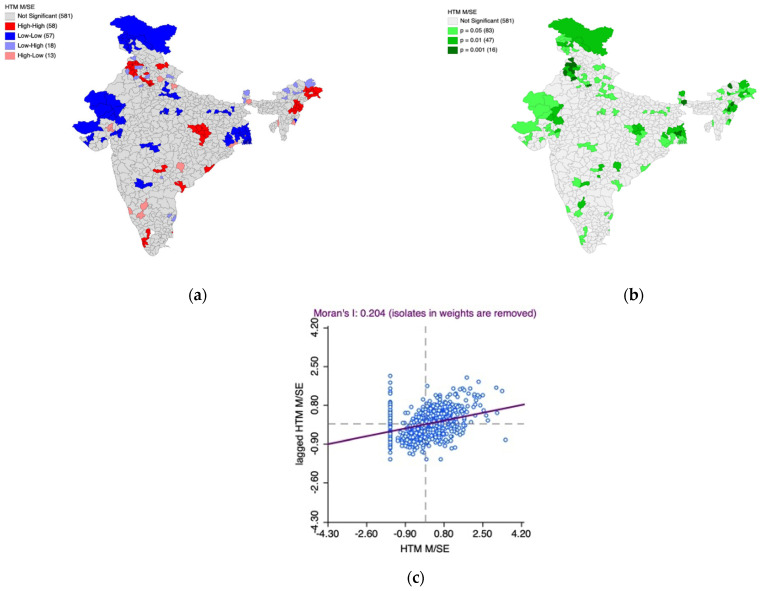
(**a**) Univariate Local Indicators of Spatial Association (LISA) cluster map for the prevalence of moderately/severely elevated blood pressure among men, (**b**) LISA significance map for the prevalence of moderately/severely elevated blood pressure among men, (**c**) Moran’s I scatter plot.

Furthermore, the Univariate Local Indicators of Spatial Association (LISA) cluster map and the LISA significance map, along with Moran’s I scatter plot, were generated to understand the underlying spatial phenomenon associated with the prevalence of moderately/severely elevated blood pressure among women (Figure 5a–c) and men (Figure 6a–c).

Based on Figure 5a–c, we observed 56 hotspot (indicated by the color red) districts scattered across the country with over 13 of them in south India. This means that there are 56 districts in the country with high prevalence of moderately/severely elevated blood pressure among women which are also surrounded by districts with the same phenomenon which is statistically significant as well. Parts of Gujarat, Rajasthan, West Bengal, and Jammu and Kashmir have most of the cold spot districts indicating that there is relatively significantly low prevalence of moderately/severely elevated blood pressure. The Moran’s I statistic (I = 0.132, *p* < 0.05) confirms the spatial autocorrelation.

Based on Figure 6a–c, we observed 58 hotspot (indicated by the color red) districts scattered across the country. This means that there are 58 districts in the country with a high prevalence of moderately/severely elevated blood pressure among men, which are also surrounded by districts with the same phenomenon, which is statistically significant. The scatter observed among hotspots also indicates high within-state heterogeneity.

In addition, most of the cold spots (indicated by the color blue) are located in Jammu and Kashmir, parts of Gujarat, Rajasthan, and West Bengal. These regions have districts with a lower prevalence of moderately/severely elevated blood pressure among men and are surrounded by the districts with the same phenomenon, which is statistically significant.

The Moran’s I statistic (I = 0.204, *p* < 0.05) confirms the spatial autocorrelation.

### 3.3. Part C: Elevated Blood Pressure

**Figure 7:**
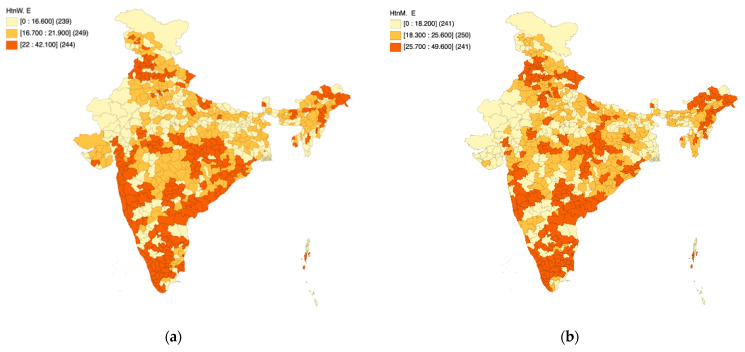
NFHS-5: District level patterns in elevated blood pressure among women (**a**) and men (**b**).

Figure 7a,b presents the spatial quantile maps for elevated blood pressure (%) across women (Figure 7a) and men (Figure 7b) as per NFHS-5 to unravel district-level patterns across the length and breadth of the country. As per the operational definition of NFHS-5, elevated blood pressure is defined as “Systolic: ≥140 mm of Hg and/or Diastolic: ≥90 mm of Hg or taking medicines to control blood pressure”. The notations “HtnW. E” and “HtnM. E” have been used on the maps to indicate elevated blood pressure among women and men, respectively. The color coding is an indicator of spatial patterns, wherein the darker colors indicate a higher percentage of men/women living with elevated blood pressure, and the lighter colors indicate a lower percentage of men/women living with elevated blood pressure.

A visual assessment of the maps reveals several dark spots in the country, indicating a higher risk of hypertension among its citizens. Over one-third (239 districts in the case of women and 241 districts in the case of men) of the districts in the country are likely to face the hypertension-induced multi-faceted public health burden.

Based on Figure 7a,b, it is observed that the district-level spatial patterns of elevated blood pressure are similar in the context of men and women. While parts of Gujarat, Rajasthan, and Jammu and Kashmir have a relatively low prevalence, the coastal districts in the peninsular region of the country experience higher prevalence (expressed in %). This phenomenon is also observed in the northeast India particularly in parts of Assam. The central part of India appears to have high within-state heterogeneity.

**Figure 8:**
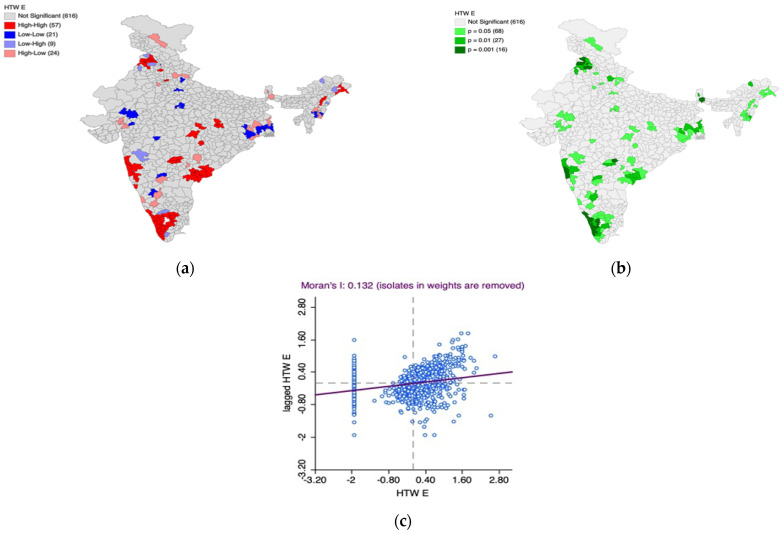
(**a**) Univariate Local Indicators of Spatial Association (LISA) cluster map for the prevalence of elevated blood pressure among women, (**b**) LISA significance map for the prevalence of elevated blood pressure among women, (**c**) Moran’s I scatter plot.

**Figure 9:**
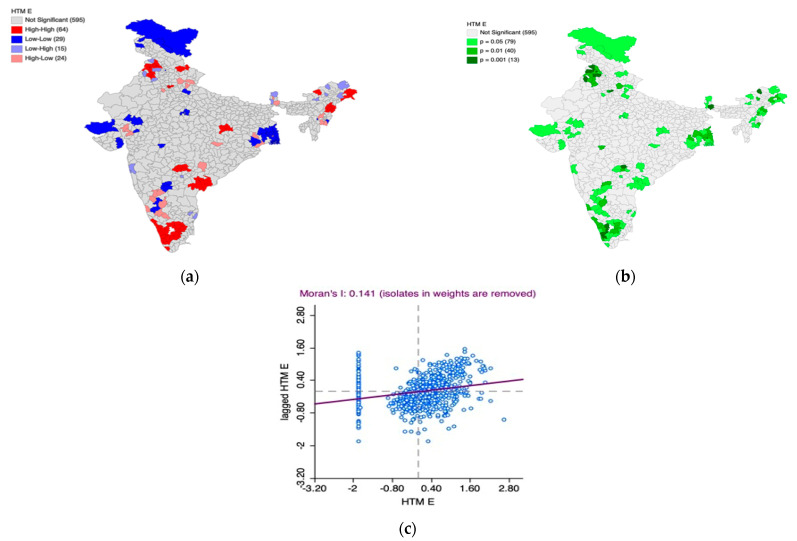
(**a**) Univariate Local Indicators of Spatial Association (LISA) cluster map for the prevalence of elevated blood pressure among men, (**b**) LISA significance map for the prevalence of elevated blood pressure among men, (**c**) Moran’s I scatter plot.

Furthermore, the Univariate Local Indicators of Spatial Association (LISA) cluster map and the LISA significance map, along with Moran’s I scatter plot, is generated to understand the underlying spatial phenomenon associated with the prevalence of elevated blood pressure among women (Figure 8a–c) and men (Figure 9a–c).

Based on Figure 8a–c, we observed 57 hotspot (indicated by the color red) districts scattered across the country with over 50% of them in South India. This means that there are 57 districts in the country with high prevalence of elevated blood pressure among women which are also surrounded by districts with the same phenomenon which is statistically significant as well. The cold spot districts (n = 21) are scattered across the country indicating high within-state heterogeneity. The Moran’s I statistic (I = 0.132, *p* < 0.05) confirms the spatial autocorrelation.

Based on Figure 9a–c, we observed 64 hotspot (indicated by the color red) districts scattered across the country. This means that there are 64 districts in the country with a high prevalence of elevated blood pressure among men, which are also surrounded by districts with the same phenomenon, which is statistically significant. Notably, all districts of Kerala but two have high prevalence of elevated pressure among men in the country.

In addition, most of the cold spots (indicated by the color blue) are located in Jammu and Kashmir, parts of Gujarat, Rajasthan, and West Bengal. These regions have districts with a lower prevalence of elevated blood pressure among men and are surrounded by the districts with the same phenomenon, which is statistically significant.

The Moran’s I statistic (I = 0.141, *p* < 0.05) confirms the spatial autocorrelation.

### 3.4. Part D: Health Insurance Coverage

**Figure 10:**
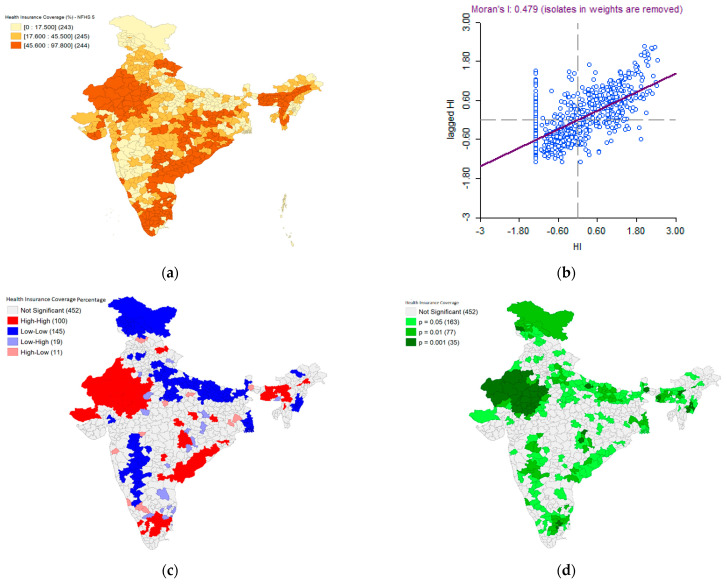
NFHS 5: (**a**) Health Insurance Coverage-District level spatial patterns, (**b**) Moran’s I scatter plot, (**c**) Univariate Local Indicators of Spatial Association (LISA) cluster map for the health insurance coverage, (**d**) LISA significance map for the health insurance coverage.

Figure 10a–d presents the spatial map indicating district-level health insurance coverage as per NFHS 5. The visualizations reveal that Rajasthan, parts of Assam, and a few districts on the eastern coast of India covering Tamil Nadu and Andhra Pradesh have emerged as hotspots whereas Jammu and Kashmir, Ladakh, parts of Uttar Pradesh, Maharashtra, and Karnataka have emerged as significant cold spots. The Moran’s I statistic (I = 0.479, *p* < 0.05) confirms the statistical significance of the spatial autocorrelation observed in the data.

### 3.5. Part E: Spatial Associations between Health Insurance Coverage

**Figure 11:**
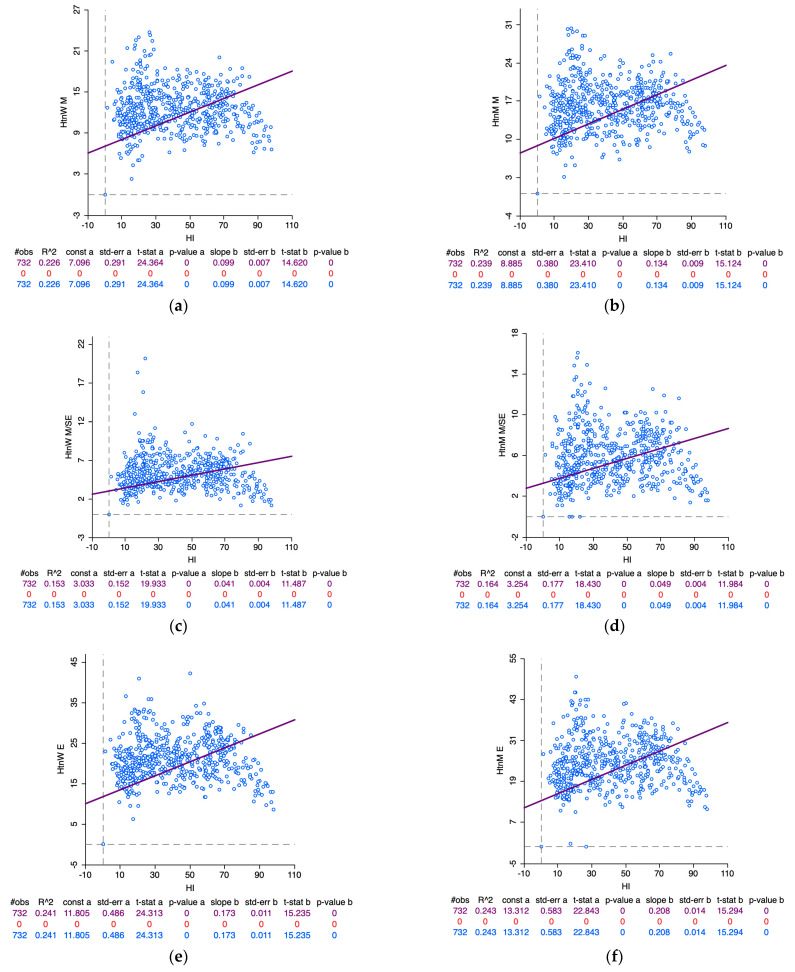
NFHS 5: (**a**) Relationship between health insurance coverage and mildly elevated blood pressure among women. (**b**) Relationship between health insurance coverage and mildly elevated blood pressure among men. (**c**) Relationship between health insurance coverage and moderately/severely elevated blood pressure among women. (**d**) Relationship between health insurance coverage and moderately/severely elevated blood pressure among men. (**e**) Relationship between health insurance coverage and elevated blood pressure among women. (**f**) Relationship between health insurance coverage and elevated blood pressure among men.

Figure 11a–f explores the relationship between health insurance coverage (%) and the following six characteristics, namely: (a) mildly elevated blood pressure among women, (b) mildly elevated blood pressure among men, (c) moderately/severely elevated blood pressure among women, (d) moderately/severely elevated blood pressure among men, (e) elevated blood pressure among women, and (f) elevated blood pressure among men. Based on an assessment of correlations incorporating the spatial components, the following statistically significant positive relationships (in the decreasing order of strength) were observed between health insurance coverage and elevated blood pressure among men (r = 0.493, *p* < 0.05); health insurance coverage and elevated blood pressure among women (r = 0.491, *p* < 0.05); health insurance coverage and mildly elevated blood pressure among men (r = 0.488, *p* < 0.05); health insurance coverage and mildly elevated blood pressure among women (r = 0.471, *p* < 0.05); health insurance coverage and moderately/severely elevated blood pressure among men (r = 0.404, *p* < 0.05); health insurance coverage and moderately/severely elevated blood pressure among women (r = 0.391, *p* < 0.05).

## 4. Discussion

In our study, we see that the coastal districts in the peninsular region of India and the districts in parts of northeastern India have the highest prevalence of elevated blood pressure. Jammu and Kashmir, parts of Gujarat, and parts of Rajasthan have a lower prevalence of elevated blood pressure. Intrastate heterogeneity in spatial patterns of elevated blood pressure is mainly seen in central India. The highest burden of elevated blood pressure is in the state of Kerala. The public health burden of elevated blood pressure is not affected by gender. Rajasthan is among the states with higher health insurance coverage and a lower prevalence of elevated blood pressure. There is a relatively low positive relationship between health insurance coverage and the prevalence of elevated blood pressure. This positive relationship seems counter-intuitive to the assumption that we set out with, which was that insurance coverage reduces barriers to effective care. One reason for this could be that health insurance in India generally does not cover outpatient care and drugs. It is also likely that higher education and higher income levels are positively associated with higher levels of insurance coverage, higher levels of stress, and higher levels of salt intake. Stress and salt intake are well known etiological factors for elevated blood pressure.

It is evident that a need exists for interventions to raise awareness of and rates of treatment of hypertension. There are significant variations in hypertension management across geographies, gender and socioeconomic strata [23,24]. This is an indication for targeted interventions for the categories with poorer metrics. Access to healthcare has been positively associated with awareness of hypertension and management of hypertension [23]. Health insurance coverage in India has been positively associated with awareness of hypertension, but not for the bottom one third of the population based on consumption per capita [23]. Health insurance in India generally covers the cost of inpatient care to the exclusion of outpatient care [25,26]. This might mean that health insurance has a limited impact in improving the diagnosis of hypertension. Access to public health centers raises the probability of adults with hypertension receiving treatment with antihypertensives. This effect is seen across all economic strata [23]. Access to public health centers has been seen to be especially significant at the poorer end of the economic spectrum. This emphasizes the critical nature of the availability of either free of cost and/or very low-cost antihypertensive medications at public health centers [23]. The health and wellness center initiative under Ayushman Bharat will play a crucial role in hypertension control in India.

A systematic review that included research from 1950 to 2013 concluded that estimates of hypertension control among adult hypertensives in India were 10.7 percent in rural populations and 20.2 percent in urban populations [10]. More recent research puts the numbers at 30.5 percent in rural populations and 33.7 percent in urban populations [23]. These data suggest that India is lagging behind more developed countries. Awareness of hypertension in England and America among men and women older than 50 years was 76 percent and 84 percent, respectively. India is more than 20 percentage points lower on the same metric [27].

The India hypertension control initiative (IHCI) has shown that the implementation of good quality hypertension treatment regimens with a drug supply that is reliable is feasible in the context of the Indian healthcare system. The IHCI has information systems that can reliably track blood pressures of patients. It can also track hypertension control rates at all three levels: facility, district, and state [4]. NCD control programs in India face several implementation challenges. This is because in several states, NCDs are not yet a high priority area [28]. Two critical barriers in the roll out and execution of initiatives such as the IHCI are poor levels of awareness in target populations and deficiencies in levels of preparedness of the health system to address the challenge [29]. One problem area is procurement of adequate medicines. Lack of quality BP monitors and insufficient manpower for NCD programs are other lacunae. Weak linkages between the community and health centers coupled with poor levels of awareness in target populations leads to poor patient retention [4]. Protocols for drugs and their doses were designed by states: Amlodipine is first line, Telmisartan is second line, and a diuretic (either hydrochlorothiazide or chlorthalidone) is third line. The WHO’s latest treatment guidelines for hypertension as well as the WHO HEARTS package protocols recommend angiotensin receptor blockers (ARBs), also known as angiotensin II receptor antagonists as the first line treatment for hypertension [30,31]. State-level hypertension experts recommended monotherapy with amlodipine for first line treatment. The reasons for this were insufficient access to lab facilities in rural geographies and also lower cost. The protocols were developed with the aim of simplifying the processes of prescription and procurement, particularly for the primary healthcare centers [4]. This is a significant step towards reducing instances of medicine stockouts at the treatment centers, which is a common concern due to well documented gaps in NCD medicine supply chains across government health facilities across India [32]. Protocols specific to drugs and dosages were designed for only three drugs. This enabled forecasting supply and stock requirements and simplified the procurement procedure. At the same time, it also decreased the unit costs associated with the medicines. This had significant positive effects on the drug stock situation [4]. Under the aegis of the HEARTS in Americas program, simple protocols for hypertension management were operationalized in the Caribbean countries and in Latin America. These protocols were found to be feasible and acceptable [33]. With the IHCI experience in the first five states being positive, treatment protocols specific for drugs and doses have been designed for more than fifteen other states in India [34]. A significant observation across all states was that blood pressure control rates were higher in the community based primary healthcare centers (PHCs). This is a clear indication that retention and control are significantly increased by decentralization of blood pressure management [4]. Adequate supply of medicines at community-based primary healthcare centers allows patients to get their refills closer to their homes, thereby reducing the need to visit government and private care providers further away [4]. Primary healthcare level interventions have yielded meaningful results in blood pressure management in Peru, Thailand, and Cuba [35,36,37].

India’s efforts at hypertension control can be aided by the continued and uninterrupted procurement of low cost generic antihypertensives by the government. This will help secure cost-free treatment for patients including their one-month refills through the established network of subcenters, Ayushman Bharat health and wellness centers, and primary health centers with trained nurses [4]. Ayushman Bharat is a national health protection program launched by the Government of India in September 2018. It has two components: 1. The Publicly Funded Health Insurance (PFHI) scheme. 2. Health and Wellness centers. The PFHI scheme is targeted at the bottom 40% of the Indian population. This amounts to about 100 million families and 500 million people, based on deprivation and occupational criteria of the socioeconomic caste census (SECC) 2011 [38]. The cashless cover is 500,000 INR (5800 EUR), which is at least 50 times the monthly earnings of more than 80% of the Indian workforce. The cover is for secondary and tertiary care hospitalization across public and private empaneled hospitals [39]. It is portable across the country. There is no cap on family size and no age bar. Pre-existing conditions are covered from day 1. Costs of diagnostics and medicines are covered up to three days pre-hospitalization and 15 days post-hospitalization. All pre-existing conditions are covered. More than 1300 procedures are covered. The scheme is administered by the National Health Authority (NHA) which reports to the Ministry of Health and Family Welfare (MoHFW). The NHA has a governing board with representatives from the central government, domain experts, and states. The financing is shared by the Central and State governments in a 60:40 ratio. Implementation can be through three models: Trust, Insurance, and Mixed. In the Trust model adopted by a majority (22/28) of the states, the scheme is directly administered by the State Health Agency (SHA) without the involvement of Insurance companies. The government bears the entire financial risk. In the Insurance model, the SHA selects an insurance company through a tendering process. The insurance company bears the financial risk and is paid a premium per family. The mixed model with both the Trust and Insurance companies is employed in brownfield states which had existing schemes.

Gaps in drug procurement systems need to be identified and plugged. Systems for accurate and timely forecasting of annual requirements need to be put in place and strengthened. Compliance to state-specific hypertension treatment protocols needs to be strengthened. This has to happen in all government healthcare facilities across the primary, secondary, and tertiary levels. Doctors need to be trained and sensitized to adjust drug dosages as required. This is especially critical in patients with uncontrolled hypertension. Telemedicine services such as e-Sanjeevani can be utilized towards this end. Patients who missed visits need to be followed up with by community health workers and auxiliary nurse midwives. Registrations and coverage can be increased by encouraging hypertension screening in the Ayushman Bharat HWCs and in the COVID-19 vaccination camps. One dedicated non-communicable disease (NCD) staff nurse must be present at every PHC. Two dedicated NCD staff nurses must be present at every community health center (CHC). Medical officer vacancies need to be filled. Opportunistic screening needs to be adopted on a large scale. Every health facility needs to have dedicated support staff to check the blood pressure of all adults visiting the facility. Validated high-quality digital BP monitors need to be used in all facilities. Information technology (IT) needs to be used to reduce missed visits. IT can help improve the quality of documentation of the visits and also send reminders to patients who miss their follow-up visits. Drug refills need to be completely decentralized to Ayushman Bharat HWCs and to sub-centers. For stable patients with controlled hypertension, drug refills can be increased from the current 1 month to 2 months. Involving the private sector can pay rich dividends.

### Limitations

The present study primarily has two objectives. Firstly, it is an attempt to understand district-level patterns of hypertension based on the data obtained from NFHS-5. While state-level patterns are usually the focus area of researchers considering the fact that policy implementations are usually carried out uniformly at state level, district-level patterns offer a micro-level understanding of the prevalence of the growing health concern in the country. The researchers would like to highlight district-level variations within the state. This will enable policymakers and healthcare providers to identify high priority districts and design interventions in a customized manner considering the socio-demographic, cultural, and environmental aspects. The study does not explore causes behind the district-level variations which provides ample scope for future work. The second objective of the study was to explore associations between district-level hypertension statistics and the recently rolled out Ayushman Bharat Program at national level. The study concludes with data-centric evidence on the relationship between hypertension and health insurance coverage. However, correlation is not causation, and this is a primary limitation of the study. The study overlooks all other contributors of hypertension and factors (political and non-political) that drive improved health insurance coverage. Additionally, the present study does not explore determinants of this relationship. The study also does not explore the effectiveness of the health insurance scheme; all of which are limitations of the present findings.

## Data Availability

Data are available on request.
